# Elimination of *Coptotermes lacteus* (Froggatt) (Blattodea: Rhinotemitidae) Colonies Using Bistrifluron Bait Applied through In-Ground Bait Stations Surrounding Mounds

**DOI:** 10.3390/insects8030098

**Published:** 2017-09-12

**Authors:** Garry Webb

**Affiliations:** Sumitomo Chemical Australia, PO Box 60, Epping, NSW 1710, Australia; gawebb@sumitomo-chem.com.au

**Keywords:** bistrifluron, *Coptotermes*, termites, termite baiting

## Abstract

The efficacy of bistrifluron termite bait was evaluated using in-ground bait stations placed around *Coptotermes lacteus* mounds in south-eastern Australia during late summer and autumn (late February to late May 2012). Four in-ground bait stations containing timber billets were placed around each of twenty mounds. Once sufficient numbers of in-ground stations were infested by termites, mounds were assigned to one of four groups (one, two, three or four 120 g bait canisters or 120 to 480 g bait in total per mound) and bait canisters installed. One mound, nominally assigned treatment with two canisters ultimately had no termite interception in any of the four in-ground stations and not treated. Eighteen of the remaining 19 colonies were eliminated by 12 weeks after bait placement, irrespective of bait quantity removed (range 43 to 480 g). Measures of colony decline—mound repair capability and internal core temperature—did not accurately reflect the colony decline, as untreated colonies showed a similar pattern of decline in both repair capability and internal mound core temperature. However, during the ensuing spring–summer period, capacity to repair the mound was restored in untreated colonies and the internal core temperature profile was similar to the previous spring–summer period which indicated that these untreated colonies remained healthy.

## 1. Introduction

Baiting is promoted as an environmentally sustainable termite management practice because it directly targets the colony and has minimal, if any, impact on non-target organisms [1,2]. Bait matrices can vary from commercially available baits in powder, pellet or solid rod form through to improvised matrices comprising paper and timber products such as cardboard, wood dust and fibre and even toilet paper [1,3,4]. Baiting relies on termites harvesting the bait and returning it to the colony for consumption either directly or through trophollaxis. Modern baiting systems rely on the use of insect growth regulators, specifically chitin synthesis inhibitors (CSIs) or benzoylphenyl urea compounds such as chlorfluazuron, hexaflumuron, novaflumuron and bistrifluron [3,5,6,7]. These active ingredients impact the chitin synthesis processes involved in moulting and other essential functions in termites.

In Australia, the most economically destructive termite in urban environments is *Coptotermes acinaciformis* (Froggatt). *Coptotermes acinaciformis* has two forms, mound-building in the tropical north and tree nesting in the south [8]. Mound building species offer the opportunity to study a discrete and confined colony [9], whereas non-mound building species are hard to locate and more difficult to treat experimentally. In southeastern Australia, *Coptotermes lacteus* (Froggatt) also builds mounds and is more geographically accessible which make it a useful model for testing treatment options for more destructive species such as *C. acinaciformis*.

There are three CSI bait active ingredients currently approved for use in Australia—chlorfluazuron, hexaflumuron, and bistrifuron. Bistrifluron is the most recently approved and the subject of the most recent studies [10,11,12]. Bistrifluron is commercially available as the Xterm^®^ Defense Against Termites baiting system. Evans [10] evaluated bistrifluron against mound-building *C. acinaciformis* in the tropical Northern Territory (Australia) whilst two studies in New South Wales (temperate south-eastern Australia) evaluated bistrifluron bait against the termitid *Nasutitermes exitiosus* (Hill) [12] and *C. lacteus* [11]. In all three studies, colony elimination was rapid (in as little as 4 weeks) and with relatively small amounts of bait required. Evans [10] used baited metal drums surrounding the mound while the two studies in southeastern Australia used in-ground bait stations placed directly into the side of mounds. While clearly demonstrating that bistrifluron bait was consumed by termites and the colonies eliminated, the method of bait delivery for *N. exitiosus* and *C. lacteus* was artificial and not representative of typical commercial baiting practices where in-ground baiting stations may be placed at some distance from the colony. In both cases, bistrifluron bait was offered to colonies directly by placing in-ground stations into the sides of mounds where termites by virtue of mound repair alone would likely contact the bait material. Further, the timing of application in both studies was during the warmer spring–summer period and may not reflect the situation during the cooler months.

During late Summer–Autumn of 2012, a further study was conducted on *C. lacteus* to evaluate the use of bistrifluron bait placed into plastic in-ground (IG) bait stations surrounding the mound in a manner that at least partially simulates commercial baiting practice. Colonies were offered various amounts of bait ranging from 120 g to 480 g in in-ground stations placed 0.5 m away from the mound so that termites would intercept the bait stations during normal subterranean foraging activities. This study was conducted largely during autumn when ambient temperatures were declining. For consistency, the same measures of colony viability used in the previous studies on *C. lacteus* [11] including mound repair capacity, internal temperature profile and visual assessment of mound characteristics were used here.

## 2. Materials and Methods

### 2.1. Field Site

The field site was in montane pasture and forest (altitude ca. 700 m) near Wildes Meadow in southern New South Wales (Australia), 150 km south of Sydney (Lat: 34.58° S Long: 150.59° E). Average annual rainfall is 1659 mm which is spread fairly evenly across the year. Average summer (January) minimum and maximum temperatures are 16 and 26 °C respectively and the average winter (July) range is 6–16 °C.

### 2.2. Test Species

*Coptotermes lacteus* is distributed along the east coast from southern Queensland to eastern Victoria forming large well insulated mounds reaching ca. 2 m in height and up to 2 m in diameter [13]. On the southern highlands of New South Wales, *C. lacteus* mounds are plentiful in pasture, forest and roadside reserves.

### 2.3. Product

Commercially available Xterm^®^ termite bait canisters (Sumitomo Chemical Australia Pty Ltd., Sydney, Australia) containing 120 g by weight of alpha-cellulose and 1% bistrifluron were used in the trial. Bait canisters were cylindrical plastic casings containing solid pellets of ca. 0.32 g each. Xterm^®^ in-ground bait stations are plastic cylindrical casings of 220 mm in length and 100 mm diameter and capped with a removable lid. Each bait station initially contained loosely packed rectangular blocks of mountain ash (*Eucalyptus regnans*) to attract termites to the bait stations. Once termites were active in stations a single 120 g bait canister was placed in on top of the timber blocks.

### 2.4. Field Installation

On 23 January 2012, twenty mounds were identified near Wildes Meadow. Four in-ground bait stations were installed ca. 0.5 m away from the outer edge of each mound and generally in the direction of the principle compass points, except where tree roots and other obstacles prevented it. Each bait station contained nine loosely fitted billets of hardwood timber 115 mm long and 15 × 20 mm in diameter which filled the bottom of the bait station. Each in-ground station was covered with a 15 × 15 cm thin plywood cover to prevent disturbance by grazing cattle and other animals.

The aim was to establish four treatment groups—one, two, three or four bait canisters (1×, 2×, 3×, 4× treatments respectively)—with five replicates each. Mounds were not pre-assigned treatments and treatment allocation depended on the natural infestation of in-ground stations. At the 1 month inspection (29 February 2012) only 54% of in-ground stations had been intercepted and so assignment of treatments was deferred. During the subsequent 7 day assessment of mound repair (6 March 2012) the number of intercepts had risen to 66%, sufficient to allow assignment of treatments. Bait canisters were placed in each infested in-ground station such that five mounds received four bait canisters, five mounds received three bait canisters, four mounds received two canisters and five mounds received one canister. One mound had no in-ground stations intercepted by 6 March 2012 and this was nominally assigned to the 2× treatment group. During the course of the study, no termites were found in any of the four in-ground station around this mound. Termites were also active in some bait stations which were not subsequently baited.

No untreated mounds were assigned from the twenty selected mounds. The one mound where in-ground stations were not intercepted during the course of the study was used as an untreated control mound, together with nine mounds used in previous trials on *C. lacteus* [11]. These mounds remained active following the cessation of the two trials reported by Webb [11] and continued to be monitored for mound temperature in the intervening period. These two earlier trials were conducted from September 2011 to January 2012 and November 2011 to March 2012. Thus all three trials were conducted across the spring to autumn period of 2011–2012 and in the same general location.

### 2.5. Assessments

Colony health was assessed using two qualitative methods: mound repair following experimental damage and monitoring of core mound temperature over time consistent with previous studies using bistrifluron bait [10,11,12]. Visual inspection of the quality of the outer casing was made at the time of mound excavation. In-ground stations were not routinely opened during the course of the trial once feeding activity had been established.

#### 2.5.1. Damage Repair

At the time of installation of the in-ground stations, a horizontal 10 cm diameter hole was cored into each mound using a hand-held auger. Core holes were positioned approximately 50 cm above ground and oriented towards the east, but subject to the presence of any obstacles. The hole was drilled until internal carton material was contacted. Carton material was usually encountered at a horizontal depth of 30–40 cm. Seven days later each hole was inspected for degree of repair and closure. Each month following installation the same process was followed to determine the degree of repair capability over time following baiting. Initially hole repair was complete in all mounds at least to the outer mud casing level. In most cases, repair exceeded this with a significant bulge on the side of the mound.

Assessment rating for damage was assessed based on the proportion of the hole repaired based on the following scale:

10%—end-wall repair only;
25%—25% hole filled with mud;
50%—50% hole filled with mud;
75%—75% hole filled with mud;
100%—100% hole filled with mud or mud repair exceeding hole.

The ten untreated mounds were similarly re-cored each month until May 2012 (the end of this trial). All ten untreated mounds were re-cored again in November 2012 and January 2013 to check the colonies were still active and to assess the degree of colony fatigue in core hole repair following the intervening winter period.

#### 2.5.2. Temperature Monitoring

A 25 mm hole was drilled horizontally into the side of each mound at a height of 50 cm and orientated towards the south. A 40 cm length of PVC tube was inserted which encased a tight fitting wooden dowel. On each end of the dowel a temperature iButton (DS1921H) (Thermodata Pty Ltd., Brisbane, Australia) was secured using electrical conduit tape, one to record internal mound temperature and the other for ambient temperature. Each end of the PVC tube was capped with a PVC cap to prevent termite entry. The inner cap was positioned in the carton material and the outer cap just on the outside of the clay casing. Data from the Thermodata buttons was downloaded using a dedicated reader and software on a regular basis. DS1921H had a lower temperature limit of 14.5 °C and maximum of 45 °C. Internal mound temperatures were recorded every 4 h from initial installation of the temperature probes on 23 January 2012 through to final excavation on 30 May 2012. All temperature probes functioned correctly for the duration of the study.

Temperature probes in the ten untreated mounds were allowed to continue logging past the end of this study to allow data to be collected over a longer period of time (Webb unpublished data).

Maximum and minimum temperatures for the nearest weather station (Moss Vale, 18 km from the study area and similar elevation) were also used.

#### 2.5.3. Final Mound Excavation

At the completion of the trial (31 May 2012), all bait stations, bait canisters and temperature probes were removed from around the treated mounds with the exception of the one 1× treated mound which still had termites present and the one mound nominally assigned 2× treatment which had no activity in the bait stations. The condition of the outer casing of the mound and the internal carton material were qualitatively assessed based on the degree of cracking and delamination of the outer casing and the condition of the carton material. One quadrant of each treated mound was excavated through to the core of the mound and below ground to determine remaining termite activity.

#### 2.5.4. Bait Removal

All bait canisters removed at completion of the trial were sealed in plastic bags and returned to the laboratory. Contents of the canisters were sieved using a 2 mm geological sieve. All termite mud was removed from pellets and fragments prior to oven drying for 48 h at 60 °C. An amount of bait (as fragments less than 2 mm in diameter) was discarded. However, it was estimated that the total weight of these fragments was no more than a few grams in total and not likely to significantly alter the final analysis of bait consumed/removed.

Samples were then weighed to determine weight of bait remaining and by inference, the amount of bait consumed or removed by termites. Bait canisters were supplied sealed in impermeable plastic and so it is assumed that moisture content when opened and installed was minimal.

### 2.6. Statistical Analysis

Mound damage repair was analysed using Kruskal-Wallis test and Dunn’s all-pairwise comparison test (Statistix 10, Analytical Software, Tallahassee, FL, USA). Similarly, bait removed relative to bait offered (as a percent value) was analysed using Kruskal-Wallis test.

## 3. Results

### 3.1. Bait Station Intercepts

**Figure 1:**
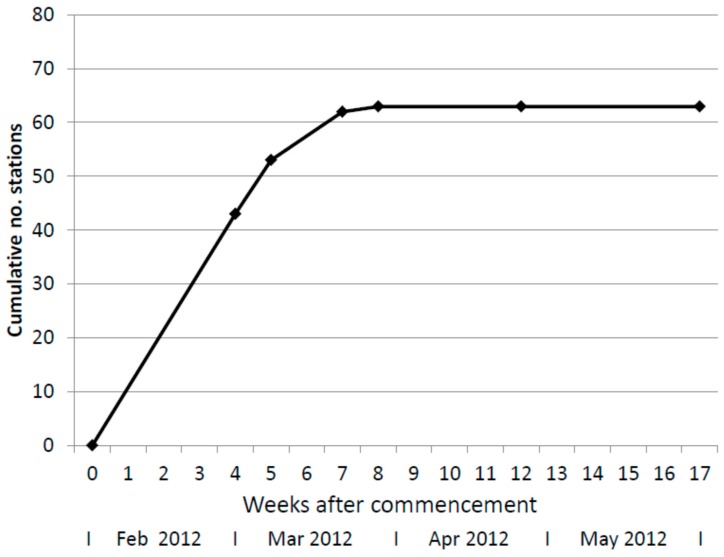
Cumulative number of in-ground bait stations infested with termites over time.

On 29 February 2012, four weeks after installation of in-ground stations, 54% had active termites feeding on the inserted timber. The following week (on 6 March 2012) a further ten in-ground stations had termite activity and on 23 March 2012 a further nine, and on 30 March 2012 one further in-ground station was occupied. After 8 weeks, 79% of all in-ground stations had active termites feeding in timbers or bait canisters and this did not increase further during the study (Figure 1).

### 3.2. Treatment Establishment

All treatments were assigned by 6 March 2012 and bait canisters installed with the exception of one 2× replicate which still had no termite activity in any of the four in-ground stations by that time and indeed still not by the end of the study. This mound was subsequently treated as a single untreated mound and combined with nine untreated mounds from the previous studies [11]. Note that while assignment of treatments was completed on 6 March 2012 termites continued to recruit to in-ground stations but no further bait canisters were added.

### 3.3. Mound Repair

**Figure 2:**
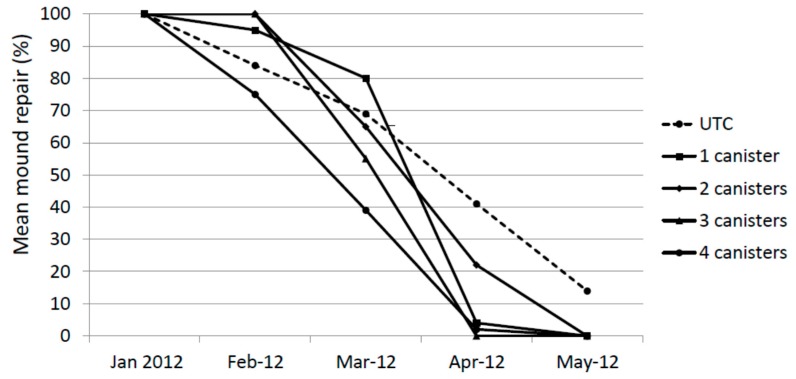
Mound repair (%) following experimental damage using a hand-held auger.

During the first assessment on 30 January 2012, 7 days after the holes were cored, all core holes were repaired completely (at least flush with the previous outer casing) (Figure 2). In many mounds the repair exceeded this.

One month later (cored 29 February and checked 6 March) the mean degree of repair in all treatments was less than 100%. This is likely to be due at least in part to the inclement weather during that 7 day period (i.e., excessively wet and cold for that time of year). However, in all cases, termites were actively working to close the hole. One month later (23 March) these holes cored on 29 February were completely repaired with the exception of one 2× mound. New holes were cored on 23 March, 23 April and 26 May and inspected one week later. The degree of repair was reduced on average in all treatments by 30 March 2012 and by 30 April 2012 almost no repair in treated mounds was evident. By 30 May 2012, there was no evidence of any repair in any treated mound. The degree of repair of untreated mounds also declined during the study, albeit at a slower pace than treated mounds. There was no statistical difference in repair for January, February and March. In April, the UTC and 2× treatments were significantly higher than the 1×, 3× and 4× treatments (Kruskal-Wallis test, F = 10.85, *p* < 0.01) and in May The UTC treatment was significantly higher than all other treatments (Kruskal-Wallis test, F = 268.3, *p* < 0.01)

**Figure 3:**
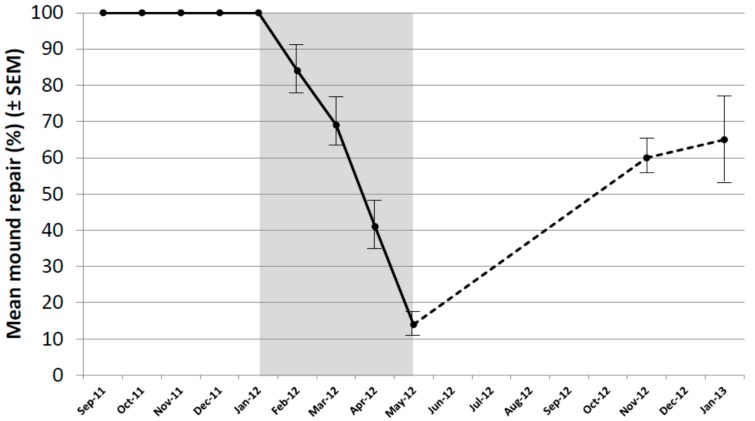
Mound repair (%) following experimental damage using a hand-held auger in untreated mounds only, covering the extended period of September 2011 to January 2013. Period of the baiting study shown in grey. Bars indicate SEM.

As a result of the decline in repair of untreated mounds, it was decided to re-core all untreated mounds in November 2012 and January 2013 (during the next autumn–summer period). The mean level of repair exceeded 60% in both months and varied from 25% to 100% (Figure 3). In all mounds, termites were actively repairing the damage in all core holes in both months indicating colonies remained healthy.

### 3.4. Mound Temperatures 

**Figure 4:**
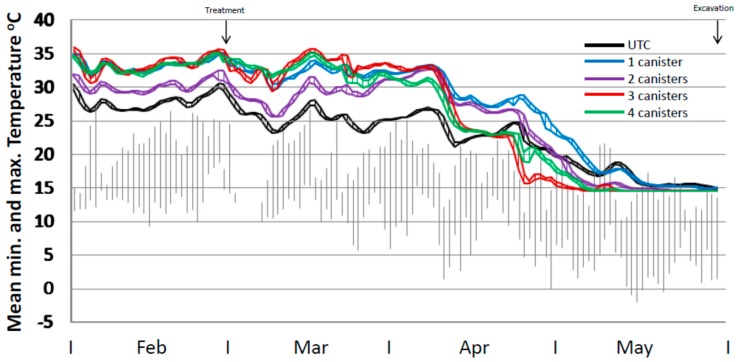
Internal temperature profile of mounds (mean minimum to maximum range) for untreated mounds (*n* = 10), and mounds treated with one (*n* = 5), two (*n* = 4), three (*n* = 5) or four (*n* = 5) 120 g canisters. Ambient temperature ranges from the closest weather station are shown in grey. Note. Temperature probes had a lower limit of 14.5 °C.

Mean temperature profiles for the four treatment regimes and the untreated control varied during the period up until treatment by up to 7 °C and during the post-treatment phase up to ca. 13 °C (end April) (Figure 4). At 7 weeks from trial commencement all treated mounds appeared to have normal temperature profiles with no decline in core temperature. This was only 3 weeks since bait placement and no effect would have been expected by then. However, mound repair assessments indicated that some retardation in repair capability was evident. Six weeks after bait placement there was a substantial drop in internal core temperature for all treatments which was also evident in the untreated mounds, coincident with a decline in ambient temperature. However, the mean temperature of the untreated mounds rose again whereas those in the treated mounds remained flat or declined. This coincided with a particularly cold period in the area. The mean temperatures for the 3× and 4× treatments continued to decline from that point until the end of the trial. For the 1× and 2× treatments the major decline in internal temperature occurred from late April onwards. All mound temperatures declined to the temperature probe lower limit of 14.5 °C by the end of May when ambient maximum temperatures were consistently below 15 °C.

**Figure 5:**
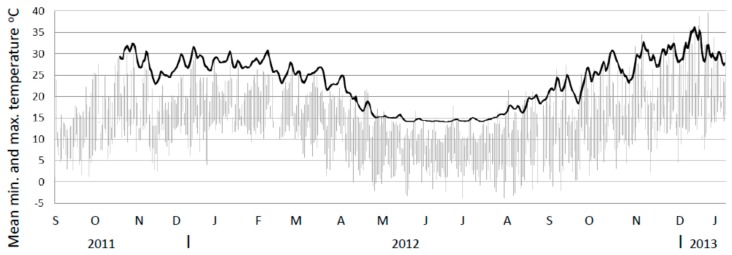
Internal temperature profile of untreated mounds (mean minimum to maximum range) for untreated mounds (*n* = 9 up till January 2012 and *n* = 10 thereafter) from November 2011 to January 2013 (black line). Note. Mound minimum and maximum temperatures are so close they appear as a single line. Ambient temperature ranges from the closest weather station shown in grey. Note. Temperature probes had a lower limit of 14.5 °C.

The mean temperature for the untreated mounds declined in a similar fashion to the treated mound towards the winter period, albeit more slowly. However, as temperatures probes were maintained in untreated mounds through to at least the next summer it provided the opportunity to investigate the response of these mounds during the post-winter period (Figure 5). Figure 5 shows temperature data for these mounds commencing in November 2011 (prior to the study) and up until January 2013. From August onwards the mean internal temperature of these untreated mounds began to increase again and by November 2012 was again similar to temperatures recorded in the previous November.

### 3.5. Final Mound Excavation

On 31 May 2012 (17 weeks after trial commencement and 12 weeks after bait placement) 18 of 20 mounds were excavated and all 18 were devoid of termite activity. The remaining two included a 2× treated mound where none of the in-ground stations had been infested and a 1× treated mound where worker activity was evident in the bait canister but not in the core hole. This mound was excavated on 5 May 2014, almost two years later, when no further termite activity was evident. The remaining 2× treated colony was still devoid of termite activity in the in-ground stations but apparently quite healthy as evident from termite presence in the final core hole, temperature profile and lack of mound deterioration.

All excavated mounds showed varying degrees of casement cracking and delamination at the time of excavation whereas untreated mounds maintained a largely intact outer casement.

### 3.6. Bait Removed by Termites

**Figure 6:**
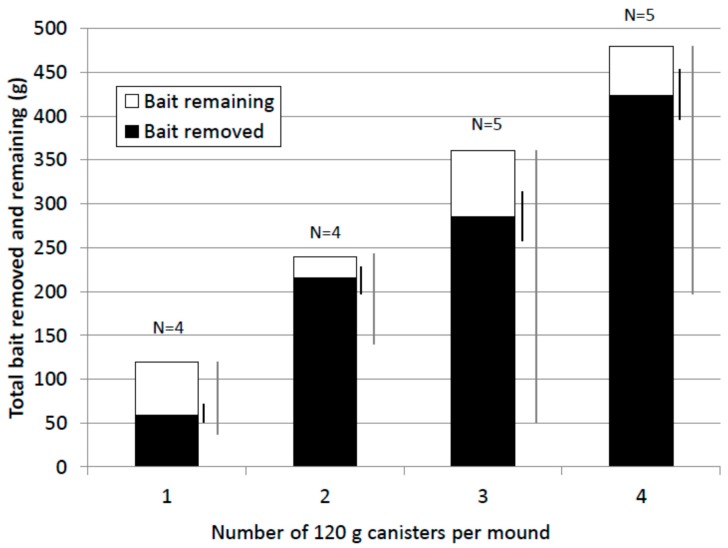
Mean bait removed by termites and bait remaining (grams). Standard Error (SEM) (black) and range bars (grey) for bait removed are shown to the right of each treatment column.

In general, termites removed a high proportion of bait offered (Figure 6). The mean value for the 1× treatment (61%) was lower than for the other treatments (90%, 86% and 88% respectively for the 2×, 3× and 4× treatments). However, percent bait removed was highly variable within and between treatments and the difference between treatments was not statistically significant (Kruskal-Wallis test, F = 1.49, *p* = 0.26). In nine of eighteen treated mounds excavated, termites removed all the bait offered (one of four 1×, three of four 2×, one of five 3× and four of five 4× treatments). Bait removed by termites ranged from 43 to 480 g. The lowest quantities of bait removed which resulted in colony elimination were 43.4 g (1× treatment), 51.5 g (3× treatment) and 61.4 g (1× treatment).

## 4. Discussion

Bistrifluron has been shown to be an effective active ingredient in termite bait products under laboratory and field conditions [10,11,12,14,15,16,17,18]. For the first time, in this study, bistrifluron bait was placed in in-ground stations positioned around *C. lacteus* mounds, simulating real-life application which relies on subterranean foraging of termites on remote food sources. Termites recruited to hardwood timber in in-ground stations relatively quickly—over 54% of in-ground stations were intercepted within 4 weeks and 79% within 8 weeks. Dhang [19] placed in-ground stations at 1 m from the centre of *Macrotermes gilvus* (Hagen) mounds and given the relative sizes of *M. gilvus* and *C. lacteus* mounds the horizontal positioning was likely to be similar. Albeit there were a small number of mounds (3) in that study, all 12 in-ground stations were intercepted by termites within 2 weeks. Evans [10] placed four 11 L steel drums containing timber and cardboard around each of 16 *C. acinaciformis* mounds and after 3 mo 97% of steel drums contained feeding termites.

In this study, 18 of 19 treated mounds were excavated 12 weeks after bait placement and all colonies were dead. The one remaining mound where termites were active at the final inspection was monitored for a further two years after the study was complete and finally declared dead when excavated in May 2014. The amount of bait required to eliminate termite colonies varies depending on the active ingredient and its concentration in the bait, speed of transfer into the colony and the method of processing and speed of consumption by termites [3,10,18,20]. In this study, all treated colonies were eliminated irrespective of the amount of bait provided. Although large amounts of bait were removed by some colonies, clearly small amounts of bait are sufficient to affect the colony. In previous studies using bistrifluron bait, the amount of bait removed varied greatly but in some cases very little bait was required to achieve elimination—as low as 25 g on *C. acinaciformis* [10], 14.3 g on *Globitermes sulphureus* [18], 6.7 g on *N. exitiosus* [12] and 7 g on *C. lacteus* [11].

This study was conducted during late summer through to the end of autumn (end of May) in a temperate climate. Previous studies with bistrifluron were conducted on tropical species [10,18] or during the spring–summer period on temperate species [11,12]. Some characteristics of colony decline were different between this study and the previous studies on *C. lacteus* [11]. All 18 excavated mounds were deemed to be dead at the time of excavation—12 weeks after bait installation. Time to elimination was similar to the previous study on *C. lacteus*. However, colony collapse metrics such as mound repair and temperature profile were harder to evaluate as the ambient temperature declined towards winter. Like treated colonies, untreated colonies also showed a decline in repair capability over the period of the study. However, experimental damage (core-holing) on these untreated mounds during the next spring–summer period (specifically November 2012 and January 2013) showed a recovery in the colonies ability to repair the mound. Similarly, temperature profiles for the untreated mounds declined towards winter together with the treated mounds making it difficult to conclude from that data alone that baiting affected the treated colonies. However post-study monitoring of the same untreated mounds showed a return to normal spring and summer temperature regimes in the mounds during the next season. *Coptotermes lacteus* is known to be tolerant of gradual seasonal change [21] and can maintain nursery temperatures 12–21 °C above ambient temperature even in winter [21,22]. French et al. [22], to demonstrate the early technology of remote temperature recording, took temperature measurements at various locations within a single *C. lacteus* mound, at a single point in time (9 a.m., 29 July 1985, mid-winter). With the exception of the probe placed in the nursery area of the mound, all temperatures above ground were ca. 7 °C and similar to the ambient temperature (6.9 °C). The temperature of the nursery area in the core of the mound was 18.9 °C. In this study, probes were placed into the carton material at 0.5 m above ground and during July in 2012 (in fact late May to early August), recorded temperatures were almost always 14.5 °C (the lower limit of the probes). The equivalent position in the study by French et al. [22] (48 cm above ground) was 7.4 °C. In fact, temperature monitoring in the years after this study indicated the lowest internal core temperature recorded across all untreated mounds was 8.5 °C and generally in the range of 9–12 °C (Webb unpublished data). The arbitrary placement of the temperature probe at 50 cm above ground probably did not intersect the nursery or maybe the nursery was moved lower as the ambient temperature declined. In *N. exitiosus* declining ambient temperature is known to bring about aggregation of termites in the nursery area to utilize metabolic heat [23,24] and these aggregations can move to different locations in the mound to optimize temperature.

There is abundant evidence that termites in general decrease their tunnelling and food retrieval activities with decreasing temperature [25,26,27,28] and may also reduce their moulting frequency [6,29,30] making them less susceptible to CSI type baits under cold conditions. For *C. lacteus* and *C. acinaciformis* there is a documented difference in foraging activity between summer and winter with aggregation in the nest occurring in winter [31,32,33]. This would suggest that baiting for termites during colder periods, particularly in temperate climates may not be as effective as during warmer periods [26]. In this study, bait placement occurred at the very end of summer when ambient temperatures were still in the 20–25 °C range (daily maximum) and did not drop consistently below 15 °C till late May (the end of autumn). All colonies removed at least enough bait during this period to cause colony decline, yet some of the measures used to assess decline (other than the final mound excavation) were less conclusive than when they were used previously during spring and summer [11]. It may be that as winter approaches and as temperature higher in the mound declines, the termite colony moves lower to warmer strata closer to the ground, and their ability or willingness to repair and maintain the upper levels of the mound decline. This might explain the reduction in mound repair observed and the decline in mound temperature at the arbitrary height above ground used. This reinforces the need for the adoption of multiple measures in determining whether termite colony elimination has occurred, particularly when termite activity is naturally limited by climatic conditions.

## 5. Conclusions

This study has confirmed the efficacy of bistrifluron termite bait when applied in in-ground stations surrounding *C. lacteus* mounds. As with the previous study on *C. lacteus*, colony elimination was not contingent on the amount of bait offered (120 to 480 g in this study). Colony elimination was achieved with as little as 43 g of bait but often termites removed the entire amount of bait offered, up to 480 g. This application was in early autumn indicating that a late season application was still efficacious. Although untreated *C. lacteus* colonies appeared to decline as winter approached using the two colony health metrics, damage repair capability and internal core temperature, monitoring of the untreated mounds in the subsequent spring–summer period indicated that mound repair capability was restored and mound internal temperature returned to normal. Colony response to declining temperature and apparent lack of activity may make it difficult to assess whether colonies have been eliminated during colder periods without destructive excavation.
